# Addresses at the Alumni Reunion

**Published:** 1899-05

**Authors:** 


### ADDRESSES AT THE ALUMNI REUNION.

*Held at the Medical Department, University of Buffalo, Feb. 22, 1899.*

### I. Address by Z. J. Lusk, M. D., of Warsaw,

President Alumni Association, Medical Department, University of Buffalo.

THIS gathering of the Alumni marks an epoch in the history of our association. By reference to the invitations you will observe that we are bidden to a reunion. A reunion it is indeed, for among the many familiar faces we see those who, for a time, have been absent from our gatherings, but who are with us again tonight, accompanied by members of the Alumni Association, which is the product of their good work and that of their associates.

Members of the Alumni Association of the Niagara Medical College: In behalf of the Alumni Association of the Medical Department of the University of Buffalo, of which I have the honor to be president, I give you greeting and a warm welcome to membership in our association. I assure you that we congratulate ourselves in having the opportunity of extending to you the honor of becoming one of us, and at the same time appreciate the honor of the privilege of extending to you the right hand of fellowship. A glance at the names of which your Alma Mater is composed, is sufficient evidence of your character and attainment. For the most part they have been added to and now form a part of the faculty of our college, a consummation that every alumnus can hail with pleasure.

Every alumnus of the Medical Department of the University of Buffalo has reason to be proud of the achievements of his Alma Mater; her history from the beginning is a record of workers; not ''learners'' but ''lifters.'' Not those who follow in the wake of scientific thought and progress, but up-to-date, and among the leaders in working out the problems of progressive medicine. From a modest beginning in an old wooden building, in 1847, her growth has been continuous, until it now occupies a home, which, from an architectural view, is one of the attractions of this great city, and as a medical institution it has no superior in this country in its perfect equipment for teaching every branch of scientific medicine. This beautiful structure with all its conveniences owes its existence to no philanthropic aid, to no endowments nor testamentary assistance, but to the indefatigable and unselfish devotion of our Alma Mater.

Now my fellow Alumni, while we contemplate with pride the great work they have accomplished, do we not often forget the relation we bear toward them? Have we displayed that appreciation and interest they have a right to expect from us? I fear that many of us are remiss in our duties, and I would remind you that this is a good time for making resolutions and would suggest that each of us here resolve to lend our best efforts to her support. Especially let us bear in mind our fraternal relations to our Alumni Association and each act our part. Let us look forward to the annual meeting as one to attend above all others.

Augmented by the addition following this reunion, our annual gathering should be one of great interest and profit. I sincerely believe in these social gatherings, such as we are enjoying tonight, and would recommend at least a semi-annual meeting to consist of a purely social character, and that the business and scientific work be relegated to the regular annual meeting.

At the proper time I may offer some suggestions for your consideration. I may, however, be pardoned for mentioning one thing which has impressed me, and that is, that some changes should be inaugurated by which to eliminate that feeling of stranger, often exhibited at our annual meetings. I have seen members come in, look around, see no familiar faces, no one to give them a word of welcome, nor the hearty handshake. After remaining a time, they go away (many times without registering) with a look of disappointment and with the fraternal impression very much weakened.

I take pleasure in announcing that this matter is being seriously considered by the executive committee, and I am sure that in the future every member will be made welcome and to feel that it is an honor to belong to the large and growing family of the Medical Department of the University of Buffalo.

### II. Address by W. G. Taylor, M. D., of Buffalo,

President Alumni Association, Medical Department, Niagara University.

This duty falls upon me not because of any special fitness, but by reason of circumstances.

It was my good fortune to be the last president of the Alumni Association of Niagara University, a position that I feel honored to hold. I am proud to be an alumnus and while we as graduates of Niagara are proud of the greater university which welcomes us as foster children, it is not without family pride that we come.

Niagara University opened her medical school fifteen years ago with a three years' course, at a time when the state law required two years only. At this time the most meager English education was considered a sufficient preliminary to the study of medicine.

Niagara University insisted on a good English education and an elementary knowledge of Latin. She adopted a graded system of study and made four annual courses of lectures obligatory. The good work done by the Niagara faculty stimulated the profession to demand of the state a law which would not only regulate the preliminary requirements of a medical student, but also his qualifications as a practitioner of medicine.

Through a technicality of this law, the graduates of the class of '92 throughout this state were declared exempt and the class of '93, desiring the same treatment as their predecessors, petitioned the legislature to delay the enforcement of this law until the following year. I am happy to say there was an exception. The class of '93 of the Niagara University, of which I was a member, considered the law just, and was willing to abide by its provisions, and we entered a protest against such an injustice and tonight the entire medical profession rejoices that this law exists and is now being enforced.

The men that we sent you this year are a type of what the Niagara University has given to the medical profession for the past fifteen years. We thank you for the consideration which you have shown us. We come here tonight with a natural feeling of regret, when we think that our Alma Mater as a distinct institution of medical teaching no longer exists, an institution whose teaching was the best and whose aims were the highest; an institution that was poor, but not to be despised; an institution hampered by selfishness and lack of money, but whose influence was far reaching and permanent. Men are influenced by circumstances, likewise institutions, and tonight we as alumni gracefully bow to the wedded state of these two medical schools, because it seems the best thing possible for the furtherance of medical teaching in this city and state. The Niagara University led in reform at a time when reform was most needed. It has served its high and noble purpose to the end and is no longer a necessity.

May the new Alma Mater of Buffalo University be as unselfish, as progressive, as desirous for the highest good of the medical profession and the community whom it serves, as the lesser school, whose work is now finished, whose passing we may regret, but of whose record we may never feel ashamed !

III. Address by WILLIAM WARREN POTTER, M. D., of Buffalo.

It is pleasant after so many years to find myself again meeting with the Alumni of the Medical Department of the University of Buffalo and participating in the ceremonies of a public reunion. It is proper for me to acknowledge the compliment paid me by the executive committee in inviting me to address you on this occasion—a compliment all the more delicate in character in that I was asked to appear as a substitute for our distinguished fellow alumnus, Dr. Peterson. I am sure we all regret his absence, all the more, too, since it is caused by illness. If he were here he would entertain and instruct you with wit and wisdom, neither of which can I expect to accomplish. However, if you will indulge me in my caprices for a few moments I will, in return, promise to be brief.

Furthermore, if you should discover in my remarks any taint of treason, or suspicion of disloyalty to my profession, my state, my country or my Alma Mater, I admonish you to give me timely and brotherly warning, that I may escape ere the coming of the dawn to the regions of the unknown, where naught is but chaos and where disloyalty and treason are not crimes.

The threshold of a new century affords a convenient pedestal from which to survey the past as well as to forecast the future. There can be found much food for serious thought in resting for a little while upon such a conspicuous eminence in the pathway of time. Looking backward, we see some work already finished and a vast amount planned and well underway. Looking forward, we observe much that awaits the laborer, many obstacles to be overcome and some prizes to be won. We may obtain valuable hints from a careful study of the past as to methods to be adopted in the building of the future. The closing gateway of the XIXth Century becomes the open door of the twentieth, which invitingly bids us enter. Before we do so let us examine for a moment some of the structures that have been erected by the officers and alumni of this great institution, for this I assume is the particular object of interest to those here assembled this evening.

When the college was established, but little more than half a century ago, there was not an educational institution above the grade of common schools on any of the territory formerly belonging to the Senecas. The self-imposed task of the founders was not easy nor was the burden light. They were, however, men strong in character, indomitable in will, learned in their profession and indefatigable in their energy. White, the proposer, came of a sturdy stock and by a fortunate marriage was enabled early to command the influence of wealth, and later to possess it himself. He provided the necessary means. Flint, the student, possessed literary accomplishments of an unusual order, which were his contribution to the common stock of the enterprise. Hamilton, the surgeon and scholar, fresh from an extended tour in Europe, brought the prestige of an already acquired fame to the new field which the establishment of this college afforded him. To this trio of sterling teachers, able physicians and sagacious men, are we indebted for the preliminary labors necessary to the establishment of the school. They were soon joined by Lee, Coventry, Webster and Hadley and it is difficult to see how seven men better fitted for the task could, at that time, have been grouped and organised for a common purpose.

Following these came a second phalanx, composed of Rochester, Moore, Dalton, Hunt and Miner, all of whom were men of the period and who bore the standard high—so high that it has never been possible to lower it. The ideals these past-masters in medical science bequeathed to their successors have ever been an inspiration—a stimulus to zeal, ambition, to do the best work possible. It is a sweet heritage and it is pleasant to know that the teachers now in control have been true to these traditions and are rendering a good account of their stewardship.

The founders were original thinkers, men of ideas, and contributed much original work to the cause of medical science. You all remember that it was in this college that vivisection was first taught in America by the masterful Dalton; that Flint here first called attention to the fact that typhoid fever was produced by the pollution of water sources with human excreta, and that he here began those studies in the niceties of diagnosis in chest diseases, that led to greater precision in internal medicine and made him famous in this branch throughout the world. It was here that clinical midwifery was first taught—an innovation in this direction that brought a maelstrom of adverse criticism upon the head of the heroic White, a storm that would have submerged a less skilful navigator, or one less stout of heart than he; here it was that Hamilton instituted those studies in regard to fractured bones that led to greater precision in treatment, with less resultant deformity, and here he wrote the first American treatise on fractures and dislocations, a classic that gave him rank with Sir Astley Cooper and Malgaigne, as an expounder of this branch of surgery. It seems proper to remind you just here that the profession of Western New York owes the memory of Hamilton a debt of gratitude for stamping out the malpractice suit evil that was growing up to such undue proportions at that time. Stimulated by designing lawyers almost every prominent doctor in this region was victimised by some poor patient to whom they had ministered for fractures or other surgical conditions. It had grown to intolerable proportions when Hamilton took up the gauntlet in behalf of the doctors, entered the courts as a witness without fee or reward and beat down the lines of attack that the lawyers had set up. He was the strongest medical witness I ever saw upon the stand, his logic was convincing, his facts incontrovertible and his tape-line was a terror to the trickery advocate. The result was that the rulings of the courts under Hamilton's influence were such as to stop these low attempts at blackmail, and it has ever since been difficult for a malpractice suit to obtain a standing in the courts of Western New York.

Finally, it must not be forgotten that it was here that the illustrious Miner first taught the world the removal of intraligamentous cysts by enucleation—a method that is discussed everywhere in relation to these tumors—and one that has indelibly stamped his name into the literature of the subject.

Mr. Chairman, these facts are not new, they are as well known to you as to me, but it is well I think to recall them at proper intervals and on suitable occasions, lest in the present environment of steam and electricity, of hurry and worry, of dazzling achievements in the present, we may overlook or forget the fact that had it not been for the work the pioneers did, the foundation they so well laid deep down in the depths, their successors of the present generation would have found it difficult—much more difficult than it has proven to be—to reach the present state of well-nigh perfected methods.

It is an interesting bit of history to recall, that with the faculty of this college originated the first move in the direction of establishing a separate state examining board. A resolution was introduced at a faculty meeting, held in February, 1864, asking the Medical Society of the State of New York to take up the question and push it to a conclusion. At a later date, viz., in 1883, the subject was again agitated, this time, too, by one of the teachers in the college, with the effect, finally, after many years of struggle with the legislature, of bringing it to definite establishment. So the Buffalo University faculty is committed to the principle by traditon and action and it could not be indifferent or half-hearted toward it if it would, and I feel sure it would not if it could. I am sure, too, that every possessor of a state license would not part with it at any price. It represents to the holder a preliminary qualification, four years of collegiate training, and a successful examination by an independent body appointed by the state—impartial, unbiased and without prejudice. It is, moreover, a passport to good professional society everywhere; and who can formulate the value of such a priceless holding?

If we turn to the work of those now responsible for the welfare of the college, we may speak in the highest commendation, though here, for obvious reasons, we must be impersonal. We have received this evening in the inspection of this great edifice and its splendidly equipped laboratories most tangible evidence of scientific research and teaching, carried on by able men, who also are not wanting in originality of methods or a forcefulness in imparting knowledge to the student. Touch a button and Prof. Cary will take you to the bedside and instruct you in all the subtleties of bronchiectasis and other diseases which afflict the chest; touch another and Profs. Mann and Van Peyma will teach you all about the complexities of maternity and the marvels in gynecic surgery; strike still another and Prof. Park will speak of the ravages of cancer and the intricate studies being carried on under his direction in the state laboratory to discover its origin and to effect if possible its prevention, incidentally he may turn out a pocket-full of appendices that he removed yesterday and explain their pathology and the surgical technique of their excision; touch still another, and Prof. Stockton will rinse out your stomach and reconstruct its coatings while you wait, or tell you how to do so for your patients as well as instruct you upon any other intricate subject pertaining to internal medicine; touch still others and Profs. Parmenter, Phelps, Pohlman and Hill will initiate you into the mysteries of anatomic, physiologic and chemic laboratories and give you the soundest instruction in those most important fundamental branches of medical science. If we could find the obscure and almost hidden ophthalmologic button of Prof. Howe, perhaps we might be able to persuade him to tell us a little of the eye and its diseases.

These, with two-score adjuncts, assistants, instructors and lecturers make up as admirable a corps of medical teachers as can be found in any college in the land. And when we contemplate that within the past year it has been reinforced by the majority of the faculty of Niagara University Medical College, it would be difficult to group or organise a more efficient teaching body.

We may with propriety pause a moment at this point to offer a few words to the Alumni of Niagara. When your Alma Mater was established there was an opportunity for excellent work on lines that Niagara adopted—viz. : a test of preliminary acquirements, three years collegiate training, and a final examination, separate and apart from the faculty. Your teachers set a most wholesome example in advanced medical standards, that, it must be confessed, was but slowly imitated by others. But when the state law went into operation, embracing all these and even more distinct advances, afterward amended to develop the present standard, it was apparent to most observers that a *raison d' etre* had ceased for the continuance of Niagara, unless she could see her way clear to advance her requirements beyond those established by law. The wisdom of your faculty can hardly be questioned in yielding to the already growing demand for a less number of medical schools and for greater facilities and stronger qualities in those that elect to remain—in other words, contraction as to number and expansion as to quality and size is the present trend.

I am sure the Alumni of Niagara will be received with open arms by those of Buffalo and that the amalgamation of the two associations, as of the two faculties, will result in a stronger single body and in stout and lasting ties among the Fellows of each. I trust that it may be found competent for the faculty of Buffalo to grant *ad enudem* degrees to the graduates of Niagara who may desire them, thus making them alumni of Buffalo in law as well as by adoption.

To return to our original thought : Century endings and century beginnings are periods of time that none of us ever saw before and we certainly shall not see them again. It becomes us therefore to look closely in retrospect into affairs and details relating to medical education, in order to gain a well-trained foresight that will guide us far into the incoming century. With the closing years of the XIXth. century came a great revolution in medical teaching. The preliminary and developmental methods incidental to a new country disappeared and newer and more detailed systems were established. The old way was not suited to our new environment and was abandoned as being inadequate to our present needs. I have before alluded to the preliminary requirements necessary to enter upon the study of medicine, the lengthened college terms, and the state examination for license. When the law separated the teaching and licensing responsibility, it released the teachers from a thraldom that was well-nigh humiliating if not degrading.

Now, released from the dreary details of the one, they can address themselves to the other with a redoubled energy. They can widen and deepen and broaden their methods and the schools will be no longer permitted to send out poorly equipped graduates. And here let me remark that the teacher of medicine in any of its branches assumes today a fearful, an awful responsibility. It is one not to be taken lightly, but it is one into which he must put his whole soul, and energy and talent, regardless, to use a current commercial phrase, "of what there is in it."

Finally, permit me to say that henceforth it will be the duty of the colleges to *teach their students medicine*—not simply to prepare them for the state examination. They must teach them so well and ground them so solidly in the science, that they will be able to take any of the higher examinations—hospital, state, civil service, army and navy—or meet any other test of fitness without fear.

Moreover, above and beyond all, they must teach them so well, that they will be adequately prepared to practise a humane profession for the relief of suffering fellow-beings bearing God's image—and this is why a medical teacher today assumes such a fearful responsibility, such an awful responsibility.

It is difficult in any assembly where scientific thinking men are congregated to refrain from some allusion to the history that has been made, whether for weal or woe, by the republic during the year 1898.

It is scarcely more than a year since the U. S. Battleship Maine was destroyed in a friendly port, and while yet on a friendly visit to a friendly power. Since that time we have raised an army of 260,000 men, fought to successful conclusion a war that demonstrated the prowess of American arms on land and sea, that has freed oppressed peoples on both sides of the globe, that has driven from the western hemisphere a nation that for 300 years and more has dominated her colonies here with amazing brutality; and, to quote Mr. Henry Watterson, "from the coming of Cortez and Pizzarro to the going of Weyler, each succeeding captain-general has seemed to emulate Alva as a rival of Satan by seeking a second immortality of damnation."

All this has been accomplished with less cost of blood and treasure and in shorter time than ever happened in the world's history, comparing expenditure and results. But who can foretell the results? It is not my desire to prophecy, for I am neither a prophet nor the son of a prophet, but it must not be forgotten that we are in the midst of a great race movement which cannot be arrested by human action or mandate. "There is a divinity that shapes our ends, rough hew them how we will." The Republic cannot retreat, it never has, it never will. It must bear its part in the regeneration and civilisation of mankind. It is marching, under God, to take its place as the first power of the world.

I deem it a fortunate circumstance that the executive committee of our association appointed this reunion on this day, when all hearts are beating time to the tune of Yankee Doodle and every soul is filled with patriotic devotion to the memory of the Father of his country. This is a day that inspires the loftiest sentiments of patriotism, and every organised society should, in my judgment, observe it in some distinctive manner by appropriate ceremony, and every American should on this day renew his vows of devotion to his country and his flag—the glorious old star spangled banner—within whose folds every citizen at home or abroad may seek and find protection.

The dawn of the new century will witness here the first exposition dedicated to the interest of all the Americas, and it is fair to expect that great benefit will result therefrom to this city and this region. Buffalo has already entered upon a new era of expansion—expansion in its best and only proper sense when applied to political economics. Its population is increasing with marvelous rapidity, its manufacturing interests are forging ahead under the stimulus of steam and electricity.

Within the radius of a night's ride from Buffalo there resides a population of 38,000,000 people. It is a city with the finest organised health department in the Union. It ought to have the cleanest streets. Within twenty-five miles of our city hall dwell more than a half million souls. Who can tell what the next century may witness? Is it too extravagant to predict that when the first census of the XXIst century is taken it will show in this same area more than 2,000,000 inhabitants?

This will mean one of the mightiest cities of the world. Let us hope that long before that time we may see Buffalo University expanded to its full compliment of departments, each complete in itself—arts, sciences, engineering, law, medicine, theology, dentistry, pharmacy, veterinary medicine—and every branch of learning known to mankind here taught to its 2,000 students, every chair amply endowed and occupied by the ablest teachers the world can produce; and proudly marching at the head of this grand educational column, will be seen with colors flying, the 1,000 students of the parent institution—the Medical Department of the University of Buffalo!

[Note by the Editor: Prof. Mann's address as chairman of the ceremonies was printed in the March edition of the Journal. These addresses are now published by request, in order to make the record complete.]

